# Evaluation of gingival phenotype: the role of gingival thickness measurements from different vertical gingival levels

**DOI:** 10.1007/s00784-024-06143-x

**Published:** 2025-01-25

**Authors:** Sude Yildirim Bolat, Muge Lutfioglu

**Affiliations:** Faculty of Dentistry, Department of Periodontology, Ondokuz Mayis University, Samsun, Turkey

**Keywords:** Gingival phenotype, Gingival thickness, Classification

## Abstract

**Objectives:**

This study aimed to accurately assess the gingival phenotype by comparing the mean gingival thickness (GT) measured at various levels with a single-point GT measurement.

**Materials and methods:**

Fifty participants were divided into thin and thick gingival phenotype groups according to two different classifications. The first classification was based on the GT measured at the base of the gingival sulcus (GT1), whereas the second classification was based on the mean of the GT (GTm) measured at the base of the gingival sulcus (GT1 point) and 1 mm apical (GT2 point) and 2 mm apical to the base of the gingival sulcus (GT3 point). The GT was measured using the transgingival method from the buccal region of 1195 teeth, including the incisors, canines, premolars, and first molars, and was statistically analyzed.

**Results:**

The mean GT was 0.95 ± 0.25 mm for GT1, 0.97 ± 0.3 mm for GT2, 0.81 ± 0.22 mm for GT3, and 0.91 ± 0.22 mm for the overall GTm. Good agreement was found between the GTm and GT1 and GT2 (k = 0.712; k = 0.758, *p* < 0.001for both), and moderate agreement was found between the GTm and GT3 (k = 0.534, *p* < 0.001). In both classifications, the effect of the dental arch location on the GT was found to be statistically significant.

**Conclusion:**

Standardized methods are required to minimize the differences in measurements from different vertical levels, which can influence gingival phenotype classification.

**Clinical trial registration:**

ClinicalTrials.gov identifier: NCT06369506.

**Clinical relevance:**

Multiple gingival thickness measurements showed that gingival phenotype varied depending on the vertical level of the gingiva measurement point. Gingival phenotype assessment based on the mean of multiple gingival thickness measurements provided precise results, emphasizing the clinical importance of multiple measurements.

## Introduction

According to the 2017 World Workshop on Classification of Periodontal and Peri-Implant Diseases and Conditions [1], “gingival phenotype” is a term used to describe the common clinical observation of variations in periodontal soft tissues, including gingival thickness (GT) and keratinized gingival width while periodontal phenotype is a term proposed to describe gingival thickness, keratinized tissue width, and buccal bone plate thickness. Using GT measurements, which involves a clinically easy and practical method, “gingival phenotypes” can be classified into two categories: thin and thick [2, 3]. 

Several studies have investigated the importance of gingival phenotype assessment to achieve the most favorable treatment outcomes and preserve gingival health. The gingival edges in the thin phenotype migrate apically during inflammation, whereas the thick phenotype involves the formation of deep pockets in response to inflammation [4, 5]. 

When evaluating the gingival phenotype, one of the most crucial factors to consider is the objective quantification of the GT, which can be measured in millimeters using a variety of techniques, including transgingival probing, cone beam computed tomography (CBCT), and ultrasonic techniques [6]. 

The gingival phenotype has been classified as “thin” or “thick” or even “very thin,” “very thick,” and “medium” in different studies [7, 8]. Researchers [7, 9, 10] have reported various millimeter cut-off values ​​for GT to distinguish the gingival phenotype. A consensus report from the World Workshop suggests that the most widely used cutoff value for differentiating GT is 1 mm [1, 11]. However, the landmark for GT measurement was not mentioned. Currently, there are large discrepancies regarding the anatomical location of the GT measurements used to determine the gingival phenotype. Additionally, it was difficult to compare the results obtained from studies using different cutoff values. To provide a different perspective on the uncertainty regarding the measurement points, GT measurements were performed at three different vertical levels, and “the mean GT (GTm)” was obtained. The primary aim of this study is to assess the consistency between mean gingival thickness (GTm) and GT measured at three individual vertical points for phenotype assessment. The secondary aim is to compare the GT measured by three-point and single-point methods across different dental arches to evaluate the applicability of using GTm for determining gingival phenotype. The hypothesis of this study is that measurement of GTm provides a reliable assessment of gingival phenotype that is comparable to measurements taken at individual vertical points.

## Materials and methods

### Study design

This was a cross-sectional study. Using the study by Collins et al. [12] to classify participants into thin and thick gingival phenotype based on GT, statistical power was found to be 80%, type 1 error rate 0.05, and minimum sample size of at least 42 individuals. Fifty participants were divided into two groups based on thin and thick gingival phenotypes using two different classifications based on measurements made from three vertical gingival levels. The first classification was based on the GT measurement taken from the base of the gingival sulcus (GT1), whereas the second was based on the GTm taken from the following three points: base of the gingival sulcus (GT1); 1 mm (GT2) and 2 mm apical to the base of the gingival sulcus (GT3). The GT was classified as thin or thick based on a cut-off value of 1 mm. Additionally, all the GT measurements were divided into six dental arch regions (maxilla/mandible anterior, maxilla right/left posterior, and mandible right/left posterior) and examined.

### Participants

The study was conducted in accordance with the principles of the Declaration of Helsinki and approved by the Ondokuz Mayis University Clinical Research Ethics Committee (OMUKAEK-Protocol No:2022/210, Clinical Trial Number: NCT06369506).

A total of 1195 teeth were investigated from participants enrolled in this study between January 2022 and August 2022. Participants, including patients and dental/hygiene students, were recruited from the University of Ondokuz Mayis. A single calibrated examiner (SY) performed all the clinical measurements. The inclusion criteria were as follows: (i) non-smokers; (ii) age ≥ 18 years; (iii) gingival health on an intact periodontium according to the 2017 World Workshop on the Classification of Periodontal and Peri-Implant Diseases and Conditions [13], (iv) no evidence of dental caries, crown shape alterations, or restorations affecting the occlusal edge of the teeth; and (v) the presence of at least one tooth representing the anterior, molar, and premolar regions in the maxillary and mandibular arches. The exclusion criteria were as follows: (i) completed or continued orthodontic treatment and use of removable dentures or orthodontic devices, (ii) use of medications that can affect the gingiva (immune system impairment, calcium channel blocker drugs, or antiepileptics), (iii) pregnancy or lactation, (iv) gingival recession, (v) history of periodontal surgery involving the maxillary and mandibular teeth, (vi) history of trauma affecting the position of the teeth, (vii) insufficiently attached keratinized gingiva (< 2 mm).

### Clinical measurements

Gingival health in the intact periodontium was determined by measuring the Loe & Silness Plaque index [14], Silness & Loe gingival index [15], bleeding on probing index [16], and probing pocket depth in six regions of each tooth in the mouth. Participants who did not meet the gingival health criteria for an intact periodontium were excluded.

**Fig. 1:**
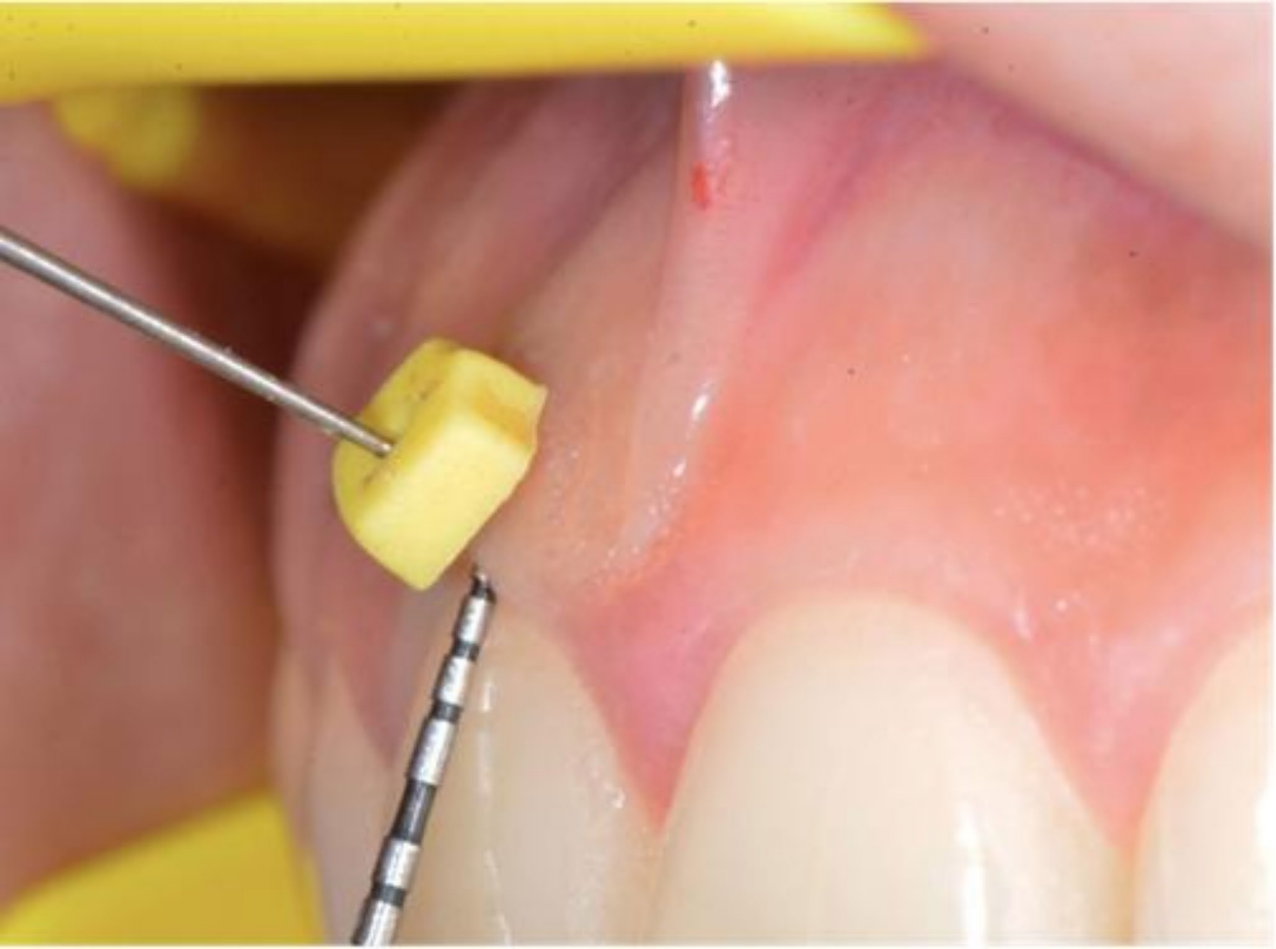
Measurement of GT using a #20 endodontic file (20 K-file; Kerr, Brea, CA, USA) by the transgingival method

**Fig. 2:**
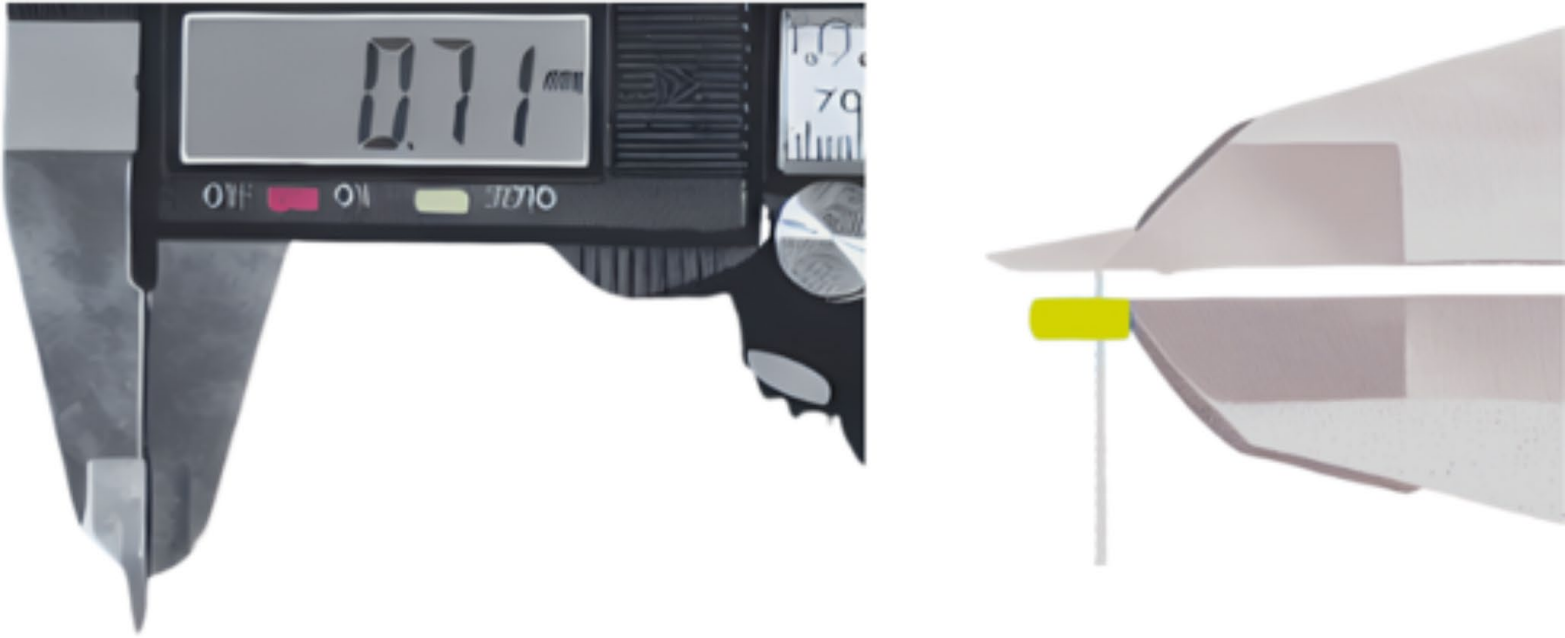
Measurement of the distance between the endodontic file and the stopper with a modified digital caliper

Before transgingival measurements, a xylocaine spray (10% lidocaine) was applied topically to reduce pain. It was measured from the buccal region of the maxillary and mandibular incisors, canines, premolars, and first molars using a #20 endodontic file (20 K-files; Kerr, Brea, CA, USA) at three different points on each tooth using the transgingival method. (Fig. 1) The first measurement was made by transgingival method after determining the point corresponding to the base of the gingival sulcus (GT1) with a 0,4 mm diameter UNC probe (Hu-Friedy, Chicago, Illinois, USA), the second measurement was made 1 mm apical (GT2) to this point, and the third measurement was made 2 mm apical (GT3) to the same point. An endodontic file with an attached plastic stopper was inserted perpendicularly into the gingiva until it reached the tooth or the bone. To prevent any undesirable movement of the plastic stopper, in accordance with the methodology of Kloukos et al., [17] a narrow new hole was drilled to keep the file fixed in place and the file was kept fixed within the stopper. The distance between the stopper and the tip of the caliper was measured using a modified digital caliper (Asimeto, Hong Kong), with an accuracy of 0.01 mm. (Fig. 2)

### Data analysis

Statistical analyses were performed using a statistical software package (IBM SPSS Statistics for Windows, Version 23, IBM Corp., Armonk, NY, USA). The agreement between the mean GT measurements and the GT1, GT2, and GT3 measurements were examined using kappa analysis. Binary logistic regression analysis was used to examine the effects of the dental arch location on the GT. Statistical significance was set at *p* < 0.05. The data are expressed as the mean ± standard deviation and median (minimum–maximum).

### Intra-examiner repeatability

All measurements were performed by a single examiner (SYB), and the intra-examiner reliability was assessed by repeating the GT measurements in 10 randomly selected patients 2 weeks later. Measurements were performed on a total of 60 teeth in the maxillary anterior region to ensure clinical accessibility and reproducibility. Accuracy and reproducibility were examined using an intraclass correlation test (ICC), and excellent agreement was observed (ICC = 0.990; *p* < 0.001).

## Results

Among the 50 participants enrolled in the study, 21 (42%) were female, and 29 (58%) were male. The age of the study participants ranged from 18 to 31 years, with a mean age of 22.42 ± 2.87 years. Five of the 50 participants had missing teeth. Therefore, 1195 teeth were measured. Measurements made using the transgingival method showed that according to the first classification (based on the GT1 point), 55.8% of teeth exhibited a gingival thick phenotype, and 44.2% exhibited a thin phenotype. According to the second classification (based on the mean GT), 37.4% of all teeth had a gingival thick phenotype, and 62.6% had a thin phenotype (Table 1). Descriptive statistics of the gingival phenotype classifications are presented in Table 1.

**Table 1:** Distribution, percentage, and descriptive statistics of gingival phenotype according to GT1 point and GTm

	First classification (based on GT1 point)			Second classification (based on GTm)			
Teeth (*n* = 1195)	Thick (*n* = 667) (55.8%)	Thin (*n* = 528) (44.2%)	*p* < 0.001		Thick (*n* = 447) (37.4%)	Thin (*n* = 748) (62.6%)	*p* < 0.001*
Mean ± SD (mm)	1,19 ± 0,13	0,78 ± 0,14			1,14 ± 0,13	0,78 ± 0,14	
Median (min-max) (mm)	1,17 (1.01–1.63)	0,78 (0.31-1.0)			1,12 (1.0-2.88)	0,8 (0.34-1.0)	

*Binary logistic regression analysis, GT: Gingival thickness, GTm: the mean GT (GT1, GT2, GT3)

The mean values of GT were 0.95 ± 0.25 mm at GT1, 0.97 ± 0.3 mm at GT2, 0.81 ± 0.22 mm at GT3, and 0.91 ± 0.22 mm at the GTm. The mean and standard deviation values for all participants are presented in Table 2.

**Table 2:** Mean, standard deviation and median values of all participants

*n* = 1195	Mean ± SD	Median (Min-Max)
GT1 (mm)	0.95 ± 0.25	0.95 (0.31–1.63)
GT2 (mm)	0.97 ± 0.3	0.97 (0.35-7)
GT3 (mm)	0.81 ± 0.22	0.79 (0.04–1.6)
GTm (mm)	0.91 ± 0.22	0.91 (0.34–2.88)

GT: Gingival thickness, SD: Standard deviation

### Evaluation of the compatibility of GT measurements at points GT1, GT2, and GT3 with GTm

Good statistical agreement was found between the GT measured from GT1, GT2, and GTm (GT1: k = 0.712; GT2: k = 0.758, *p* < 0.001). Moderate agreement was found between the GT3 points and GTm (k = 0.534; *p* < 0.001) (Table 3).

**Table 3:** The gingival phenotype distributions and percentages of GT1, GT2 and GT3 points in 1195 teeth showing the compatibility of GT1, GT2 and GT3 points with the GTm

	GTm	
	k	p
GT1 point	0.712	< 0.001*
GT2 point	0.758	< 0.001
GT3 point	0.534	< 0.001

*Kappa analysis, GT: Gingival thickness

### Comparison of gingival phenotype distributions of dental arch regions

In the analysis of the first classification according to GT1, multivariate analysis revealed a thicker GT in the maxillary right posterior region than in the maxillary anterior region (odds ratio [OR] = 0.513; 0.019), mandibular anterior region (OR = 0.38; *p* = 0.002), or mandibular left posterior region (OR = 0.454, *p* = 0.013). When examined univariately, no statistically significant difference was detected between the dental arch regions in terms of GT (*p* > 0.050). No statistically significant differences were detected between the maxillary and mandibular anterior regions or between the anterior and posterior regions (*p* > 0.050) (Table 4).

**Table 4:** Distribution and percentages of different dental arch regions according to phenotype classification, examination of the effect of different dental arch regions on GT (according to the classification based on GT1 point)

Arc location	GT *n* = 1195		Univariate		Multiple			
	Thick (*n* = 667)	Thin (*n* = 528)	OR (%95 CI)	p	OR (%95 CI)	p		
Maxilla right posterior (%)		84 (56.3)	65 (43.7)	Reference				
Maxilla anterior (%)		168 (56)	132 (44)	1.027 (0.692–1.526)	0.893	0.513 (0.294–0.896)	0.019*	
Maxilla left posterior (%)		84 (56)	66 (44)	1.027 (0.651–1.622)	0.907	0.854 (0.471–1.549)	0.605	
Mandible right posterior (%)		82 (55,4)	66 (44,6)	1.027 (0.651–1.622)	0.907	0.701 (0.405–1.213)	0.204	
Mandible anterior (%)		168 (56)	132 (44)	1.027 (0.692–1.526)	0.893	0.38 (0.206–0.698)	0.002	
Mandible left posterior (%)		81 (54.7)	67 (45.3)	1.056 (0.669–1.665)	0.816	0.454 (0.242–0.849)	0.013	

*Binary logistics reg. analysis; GT: Gingival thickness; OR: Odds ratio; CI: Confidence interval Cox&Snell R2 = 22.5%; Nagelkerke R2 = 30.1%

We examined second classification according to the mean GT of the three points (GT1, GT2, and GT3) using multivariate analysis. There was a thicker GT in the right maxillary posterior region than in the maxillary anterior region (OR = 2.674; *p* < 0.001), mandibular anterior region (OR = 10.0270; *p* < 0.001), and left mandibular posterior region (OR = 1.975; *p* = 0.045). Univariate analysis revealed a thicker GT in the right maxillary posterior region than the maxillary anterior region (OR = 2.688; *p* < 0.001), mandibular anterior region (OR = 45.789; *p* < 0.001), left mandibular posterior region (OR = 2.01; *p* = 0.045), and right mandibular posterior region (OR = 1.667; *p* = 0.03) (Table 5).

**Table 5:** Distribution and percentages of different dental arch regions according to phenotype classification, examination of the effect of different dental arch regions on GT (according to the classification based on the GTm)

Arc location	GT (*n* = 1195)		Univariate		Multiple	
	Thick (*n* = 447)	Thin (*n* = 748)	OR (%95 CI)	*p*	OR (%95 CI)	*p*
Maxilla right posterior (%)	90 (61.2)	57 (38.8)	Reference			
Maxilla anterior (%)	111 (37)	189 (63)	2.688 (1.791–4.036)	< 0.001	2.674 (1.633–4.379)	< 0.001*
Maxilla left posterior (%)	98 (65.3)	52 (34.7)	0.838 (0.522–1.344)	0.463	0.703 (0.389–1.27)	0.243
Mandible right posterior (%)	72 (48.6)	76 (51.4)	1.667 (1.049–2.647)	0.030	1.234 (0.701–2.172)	0.466
Mandible anterior (%)	10 (3.3)	290 (96.7)	45.789 (22.459–93.356)	< 0.001	10.027 (4.34- 23.164)	< 0.001
Mandible left posterior (%)	66 (44)	84 (56)	2.01 (1.265–3.192)	0.003	1.975 (1.014–3.847)	0.045

*Binary logistic regression analysis; GT: Gingival thickness; OR: Odds ratio; Cl: Confidence interval confidence interval, Cox & Snell r2 = 33.1%; Nagelkerke r2 = 46.1%

## Discussion

Accurate assessment of gingival phenotype is crucial for successful restoration, implantation, and orthodontic treatment. Available evidence indicates that individuals with thin and narrow gingiva tend to have more recessions than those with thick and wide gingiva [18]. A flap thickness of 0.8–1.2 mm increases predictability in root coverage procedures. Initial GT is an important factor for success [18, 19]. For this reason, clinical measurement of GT is important in the evaluation of gingival phenotype.

Transgingival probing is a widely used and relatively simple method for measuring GT by inserting a dental instrument, such as an endodontic file, perpendicularly into the buccal gingiva [9]. Compared with transgingival probing, CBCT is a relatively reliable method in the anterior and posterior regions, whereas the ultrasound method will only provide limited accuracy in the posterior regions due to the difficulty of accessing the posterior regions, according to a recent systematic review [20] that examined methods for diagnosing gingival phenotypes by quantitatively assessing GT. Due to the presence of radiation in the CBCT method, the need for a high-cost device, and the training required to perform X-ray measurements, the transgingival probing method was used in this study because it is relatively simple and widely available. However, since this technique has been previously reported [9] to typically require local anesthesia, which may cause patient discomfort and a temporary increase in local volume, topical anesthesia was used in this study.

While some classifications differentiate GP into simple categories like ‘thin’ and ‘thick,’ others use more detailed classifications, subdividing GP based on additional morphometric features. This study followed the dichotomous classification as recommended by the 2017 World Workshop [1], aiming to standardize and simplify the approach. Previously, Müller et al. [21] measured GT at the base of the gingival sulcus with the ultrasonic method and classified it as “thin narrow”, “thin wide” and “thick wide”, while De Rouck et al. [22] classified the gingival phenotypes as “thin scalloped”, “thick flat” and “thick scalloped” with the translucency method. Kloukos et al. [17] defined the groups as “thin”, “medium”, “thick” and “very thick” by measuring 2 mm apical to the free gingiva. This study contributed to the literature by focusing on the ideal measurement point with a different approach. Achieving consensus on this issue in future research may allow for more consistent classifications and increase comparability across studies.

The diagnostic reliability and repeatability of the GT measurements may have been influenced by the anatomical point chosen for the measurement [20]. The lack of a consensus and standardized method for determining the gingival phenotype may be due to the fact that GT measurement is performed at different points. Rodrigues et al., [11] who examined the GT at different anatomical landmarks in the maxillary anterior region, suggested a 2 mm apical to the gingival margin. However, there is uncertainty regarding the full dental arch. To address this, our study used GT measurements at three points up to the first molars in the upper and lower jaws: GT1 (base of the gingival sulcus), GT2 (1 mm apical to GT1), and GT3 (2 mm apical to GT1). Various anatomical points, including the base of the gingival sulcus [7, 23], gingival margin [24–26], cemento-enamel junction [27, 28], mucogingival junction [29, 30], and alveolar crest [26, 27] have been used in previous studies. Additionally, measurements at the mesial, mid, and distal gingival points were documented [31]. The findings of this study were 0.95 ± 0.25 mm at GT1, 0.97 ± 0.3 mm at GT2, and 0.81 ± 0.22 mm at GT3 and revealed a significant difference in the GT distribution at each point, indicating that GT values vary at different points, consistent with previous studies [27, 28, 32, 33]. Aside from most studies in the literature, which have been conducted in the maxillary anterior region, a recent study, like ours, performed measurements at multiple-points in both the maxilla and mandible, extending to the premolars. Zhao et al. [28] measured gingival thickness (GT) at four points from the gingival margin to the alveolar crest: 0.81 mm at GT1, 0.99 mm at GT2, 1.25 mm at GT3, and 0.58 mm at GT4. Although the measurement levels differ from ours, similar to our results, the highest mean GT was observed at GT2, while the lowest was at GT3. However, these anatomical levels are still different from each other, and it is clear that standard reference points are necessary for a precise comparison of these results. A systematic review [11] focused on determining the ideal point for GT measurement in periodontally healthy maxillary anterior teeth reported a wide variation in GT with high heterogeneity. This makes it difficult to determine the ideal anatomical location. Therefore, it is difficult to compare the methods and findings reported in this study with those of similar studies [27, 28, 32, 33]. Although their methods and findings differed from those of our study, Pascual et al. [32] measured the GT up to 1 mm apical to the gingival sulcus and found comparable results between the maxillary and mandibular teeth. This may correspond to GT2 in the current study. Stein et al. [33] evaluated GT at six points and observed significant differences. The authors [33] reported that the apical point of the gingival margin at 1 mm is not the best point to evaluate the GT but may be useful in restorative and prosthetic planning to prevent underlying reflection of restorative materials. It should be noted that both this study and previous studies were heterogeneous, with different measurement methods, measurement points, and dynamically changing phenotype classifications affecting the results. These results also suggest that the selection of vertical levels from the gingival margin to the mucogingival junction should be based on their functionality for diagnosis and treatment planning. Future studies evaluating the clinical significance of each vertical level may provide valuable insights.

Gingival thickness measurements at different anatomical points may lead to variations in the gingival phenotype classifications. We classified the participants into “thin” and “thick” gingival phenotypes based on GT measurements at GT1 and GTm. The distributions of “thin” and thick” gingival phenotypes according to the GTm and the 1 mm cut-off value recommended at the 2017 World Workshop [1] at each vertical level was compared using kappa analysis. The agreement between the GTm and GT1 measurements was 83.3%, and 82.1% with GT2, and a statistically significant level of agreement was observed between them. However, a moderate level of compatibility was observed with GT3. Our findings showed that GTm was consistent with GT1, GT2, and GT3 in gingival phenotype classification. The compatibility of GT1 and GT2 with GTm was higher than that with GT3. This may indicate that GT1 and GT2 have more compatible results than those with GT3, and can be used to determine the gingival phenotype and treatment plan.

The “thin” and “thick” groups of the GT1 and GTm-based classifications were examined, and there were statistically significant differences between the “thick” and “thin” groups in both methods. The GTm-based classification classified a higher percentage of teeth as “thin” compared to GT1. This suggested that calculating the mean GT across multiple points was a more stringent classification for the “thick” phenotype. However, the mean values ​​of the groups were close to each other in both classifications. It is thought that the GTm-based classification may represent the general gingival phenotype variability and is potentially reliable for comprehensive assessments. Since there is no study on GTm in the literature, the results obtained could not be directly compared. These results should be supported by similar studies in the future.

Various studies [21, 27, 29, 32, 34, 35] have investigated whether the gingival phenotype exhibits specific differences across the locations of the dental arch. Although some studies [27, 36] have found significant differences between the anterior arches of the lower and upper jaws, our analysis revealed no significant differences in the anterior arches according to the two classifications. This finding aligns with those of Kurien et al. [35] who similarly found no differences. However, in both classifications, the maxillary posterior region exhibited a thicker GT than that of other regions. Lee et al., [29] who assessed up to the molar teeth, also noted an increase in the GT from anterior to posterior. Kolte et al. [36] reported differing GTs between the anterior maxilla and mandible, emphasizing regional differences. Similar trends were observed in participants categorized as GT1 and GTm when evaluating the effects of sex and dental arch location on GT. Specifically, the maxillary posterior arch exhibited a thicker GT than that of other regions, with no significant differences between the anterior maxilla and mandible. Similar results were obtained in the two analyses performed with classifications based on mean GT. Another important point is that in the binary logistic regression analysis performed according to the mean GT, the effect of the dental arch location on the GT was statistically more significant. More sensitive results were obtained when the ORs were examined. Although this is not a practical method in clinical practice, it may be useful in providing more sensitive results in the evaluation of the relationship between multiple clinical and morphological parameters and GT. However, as mentioned previously, no study has been conducted on mean GT, and these results need to be confirmed.

This study has several limitations in terms of potential measurement errors due to the use of the transgingival probing method. Future studies may benefit from alternative methods, such as CBCT, to minimize measurement errors, and parameters such as tooth morphology and keratinized gingival width may be included. In addition, given that the age range of the participants in this study was limited, and they were selected from a specific geographical region, these findings should be generalized with caution.

## Conclusion

This study presented a detailed analysis by including the entire dental arch down to the first molars and measuring it at three points on each tooth and showed that GT may vary according to the location of the dental arch. Although the three-point measurement is not practical, the good agreement of the GTm with points GT1 and GT2 and the similar results of the GTm with GT1 in showing GT changes in different dental arches indicate that the GTm can also be used for gingival phenotype evaluation. In conclusion, the GT and gingival phenotypes vary at different vertical levels of the gingiva, highlighting the clinical importance of the three-point measurements.

## Data Availability

No datasets were generated or analysed during the current study.
